# Virus-induced overexpression of heterologous *FLOWERING LOCUS T* for efficient speed breeding in tomato

**DOI:** 10.1093/jxb/erad369

**Published:** 2023-10-03

**Authors:** Yingtian Deng, Antonia Yarur-Thys, David C Baulcombe

**Affiliations:** Department of Plant Sciences, University of Cambridge, Downing Street, Cambridge CB2 3EA, UK; National Key Laboratory for Germplasm Innovation and Utilization for Fruit and Vegetable Horticultural Crops, College of Horticulture and Forestry Sciences, Huazhong Agricultural University, Wuhan, 430070, China; Department of Plant Sciences, University of Cambridge, Downing Street, Cambridge CB2 3EA, UK; Department of Plant Sciences, University of Cambridge, Downing Street, Cambridge CB2 3EA, UK; University College Dublin, Ireland

**Keywords:** Flowering, FT, potato virus X, RNA silencing, speed breeding, virus vector

## Abstract

Potato virus X (PVX) vectors expressing the *Arabidopsis thaliana FLOWERING LOCUS T* (*FT*) or tomato *FT* ortholog *SINGLE-FLOWER TRUSS* (*SFT*) shortened the generation time in tomato due to accelerated tomato flowering and ripening by 14–21 d, and caused a 2–3-fold increase in the number of flowers and fruits, compared with non-infected or empty vector-infected plants. The Arabidopsis FT was more effective than the tomato orthologue SFT and there was no alteration of the flower or fruit morphology. The virus was not transmitted to the next generation; therefore viral vectors with expression of a heterologous FT will be a useful approach to speed breeding in tomato and other species.

## Teaser

Potato virus X-induced overexpression of Arabidopsis *FLOWERING LOCUS T* (*FT* ) and tomato *SINGLE-FLOWER TRUSS* (*SFT* ) accelerated tomato flowering and ripening, with FT being more effective than the endogenous SFT.

## Introduction

Tomato is an a widely cultivated South American crop that is also a model species for basic research because it is informative about aspects of growth and development that cannot be addressed using Arabidopsis—the standard model in plant biology. It is useful, for example, for investigation of fleshy-fruit formation (Kimura and Sinha, 2008) and patterns of branching during vegetative development (Xu *et al.*, 2015). A limitation of tomato, however, is the long generation time. It is typically 65–100 d and a system for speed breeding (Wanga *et al.*, 2021) of tomato would, therefore, benefit both basic and applied research.

One approach to speed breeding exploits the FLOWERING LOCUS T (FT) regulator of floral transition (Koornneef, 1991; Kardailsky *et al.*, 1999; Kobayashi *et al.*, 1999). The FT protein is transported through the vasculature to the floral meristem where it acts together with its activator CONSTANS (CO; Takada and Goto, 2003; An *et al.*, 2004; Corbesier *et al.*, 2007). The role of FT as a mobile inducer of flowering is conserved in a wide range of plants (Lin *et al.*, 2007; Tamaki *et al.*, 2007; Krieger *et al.*, 2010; Abelenda *et al.*, 2016) and its transgenic expression stimulates early flowering and reduces generation time (Lewis and Kernodle, 2009; Wigge, 2011).

An alternative, non-transgenic method of speed breeding uses viruses as expression vectors. For use of such vectors a gene of interest is inserted into the vector at a site that does not interfere with viral genes and it is expressed, often at very high levels, in the infected plant. This virus-mediated overexpression (VOX) allows tracking of virus infection in the plant (Abrahamian *et al.*, 2020) with reporter genes and it is useful for functional analysis of plant genes (Lindbo *et al.*, 2001; Manning *et al.*, 2010; Lee *et al.*, 2012; Zhang *et al.*, 2013; Majer *et al.*, 2017). VOX of *FT* results in accelerated virus-induced flowering (VIF) in crop breeding programs (McGarry *et al.*, 2017) and research. It has advantages over transgenic *FT* expression or the use of rapid cycling varieties including microTom (Marti *et al.*, 2006) because it can be deployed on multiple cultivars without the need for multiple transformations or crossing.

Potato virus X (PVX) is a well-established virus vector with potential in VIF. This vector is infectious on a broad range of *Solanaceous* plants and it was initially developed for VOX (Chapman *et al.*, 1992), but has been used widely for virus-induced gene silencing (VIGS; Ruiz *et al.*, 1998; Lu *et al.*, 2003). PVX-based VOX of *FT* in tobacco was used in a mutation analysis of FT function (Qin *et al.*, 2017) but it has not been tested previously as a system for speed breeding of tomato.

In this study, we tested PVX as an alternative to tobacco rattle virus (TRV) or tobacco mosaic virus as a vector of VOX of *FT* in tomato (*Solanum lycopersicum*, *Sly*). We also compared the tomato and *Arabidopsis thaliana* homologues to accelerate flowering. We hypothesized that, with the tomato insert, there would be VOX of the *SINGLE FLOWER TRUSS* (*SFT)* from the viral genome superimposed on VIGS of the endogenous *SFT.* With the Arabidopsis *FT,* in contrast, the VIGS of the endogenous sequence would be avoided due to the sequence heterogeneity between the two sequences and the infected plant would express both the viral and endogenous genes.

Our findings validate the previously demonstrated use of VIF as an approach to speed breeding. We also illustrate how the overexpression of *FT* was more effective than *SFT*, most likely because RNA silencing of the endogenous gene was minimized. Our findings confirm the potential for PVX-mediated expression of *FT* as a convenient and efficient system for speed breeding in tomato and indicate that heterologous *FT* genes may be more effective than those from the species being infected.

## Materials and methods

### Plant materials and growth conditions

Tomato ‘M82’ (*Solanum lycopersicum*), *Nicotiana benthamiana*, and Arabidopsis (Col-0) plants were grown in soil in the plant growth facility under long-day conditions (light/dark: 16 h/8 h, 300 µmol m^–2^ s^–1^) at a temperature of 25 °C with 65–70% humidity, before and after inoculation.

### Recombination with PVX vectors

The PVX constitutive expression vector pGR107 has been described previously (Lu *et al.*, 2003). For construction of PVX vectors, we inserted PCR-amplified 325 bp cDNA fragments of the *SlyPDS* (*Phytoene desaturase*; Solyc03g123760.3.1, ITAG4.0) and full length insert of *SFT* (534 bp, Solyc03g063100.2.1, ITAG4.0) and *FT* (AT1G65480, 528 bp, Araport11) into a *Sma*I-cut pGR107 vector. Sequences and primers used in these constructions are listed in Supplementary Table S1.

### Tomato inoculation

To prepare the inoculum, we used overnight LB liquid medium cultures of *Agrobacterium tumefaciens* (strain GV3101) transformed with the above empty or assembled vectors using an electroporation system (BioRad Gene Pulser Xcell^TM^, BioRad, USA). After centrifugation and resuspension of the cells in infiltration medium (10 mM MES, 200 μM acetosyringone) at an OD_600_ =0.1 we infiltrated tomato cotyledons (14 days after germination, DAG) by using a needleless syringe. There were least four replicate plants for each treatment and we repeated the assays three times or more as indicated in the legend of each figure.

### RNA extraction and RT–PCR analysis

Total RNA was extracted from tomato and *Nicotiana benthamiana* leaves 21 days after infiltration (DAI) and 28 DAI from Arabidopsis leaves by using Trizol reagent (Invitrogen^TM^, USA). First-strand cDNA synthesis used 2 μg total RNA with random primers by using RevertAid First Strand cDNA Synthesis Kit (Invitrogen^TM^, USA) and RT–PCR used the DreamTaq Green DNA Polymerase (Thermo Scientific™, USA) before fractionation of the product using a 1.2% agarose gel. Primers used in the RT–PCR are listed in Supplementary Table S1.

### Reverse transcription—quantitative PCR (qRT–PCR) analysis

qRT–PCR used the Luna® Universal qPCR Master Mix (NEB, USA) in a 10 μl total sample volume [5 μl of 2× Luna® Universal qPCR Master Mix, 1.0 μl of primers (10 µM), 1.0 μl of cDNA (200 ng µl^–1^), and 3 μl of distilled, deionized water]. We calculated relative gene expression values using the 2^−ΔΔCt^ method with tomato *Tubulin* gene (Solyc04g081490.3.1, ITAG4.0) and *SKP1* gene (*S-phase kinase-related protein 1*; Solyc01g111650.3.1, ITAG4.0) as internal reference genes. There were independent samplings of at least two biological replicates and three technical replicates were included for each treatment. Primers used in the qRT–PCR are listed in Supplementary Table S1.

### Data analysis

All the statistical analyses in this study used the Student’s *t*-test method. The histograms and box graphs were generated via GraphPad Prism 9.5.0 (GraphPad Software, San Diego, CA, USA).

## Results and discussion

### PVX-mediated gene silencing and expression in tomato

**Fig.1.**
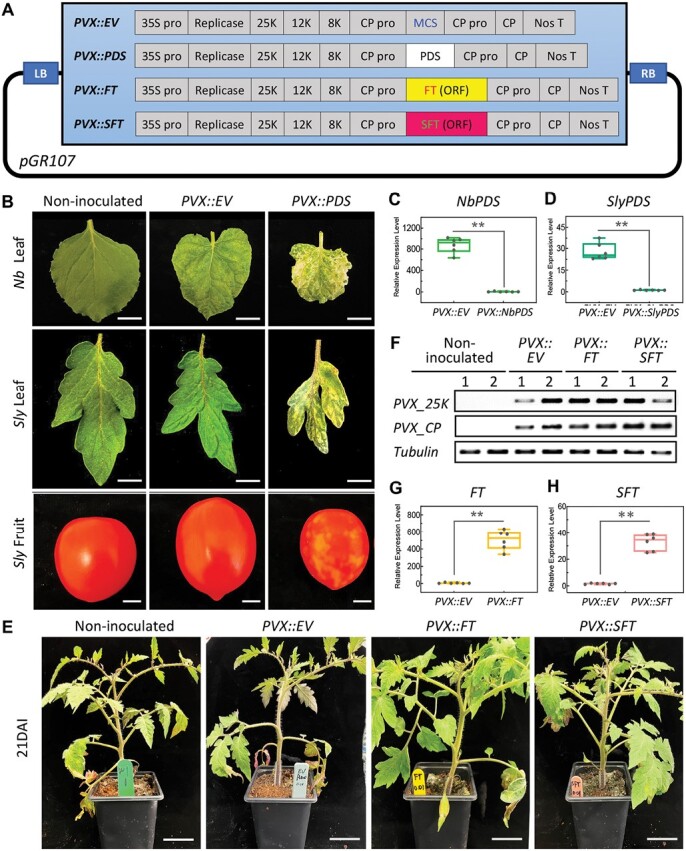
PVX vectors and *PVX*-induced VIGS and VOX in tomato. (A) Schematic diagram of PVX vector components. (B) *PVX*-induced *PDS* silencing phenotypes in *N. benthamiana* (*Nb*) leaves, tomato (*Sly*) leaves and fruit. Scale bar=1 cm. Four *N. benthamiana* plants and four tomato plants were used in the experiment; the experiment was repeated three times. The control vector used was *PVX::Empty* (EV, pGR107). (C, D) Relative expression level of *PDS* in *PVX::EV-* and *PVX::PDS-*inoculated (C) tobacco and (D) tomato leaves at 21 DAI (**P<0.01, *n*=6). The box plot shows minimum to maximum with all points. (E) Tomato plants inoculated and non-inoculated with PVX vectors at 21 DAI. Scale bar=2 cm; eight tomato plants were used for each group and the experiment was repeated three times. (F) Abundance of PVX coat protein (PVX_CP) and 25K RNA (25K movement protein) in tomato plants from (E) determined by RT–PCR, each group has two biological replicates (1 and 2). (G) Relative expression level of *FT* in *PVX::EV* and *PVX::FT*-treated tomato leaves at 21 DAI determined by qRT–PCR; expression values are relative to reference genes (**P<0.01, Student’s *t*-test; *n*=6). The box plot shows minimum to maximum with all points. (H) Relative expression level of *SFT* in *PVX::EV* and *PVX::SFT*-treated tomato leaves at 21 DAI determined by qRT–PCR (**P<0.01, Student’s *t*-test; *n*=6). The box plot shows minimum to maximum with all points.

To test the PVX system for VIF, we used PVX vectors with the intact ORF of *FT* from tomato and Arabidopsis. The tomato and Arabidopsis FT homologues have 73.6% and 69.3% identity at the protein and nucleic acid levels, respectively (Supplementary Fig. S1). We also constructed vectors for VIGS with fragments of *PDS* from tomato and *N. benthamiana* (Nb) cDNA. The *PVX::PDS* constructs were introduced to induce VIGS-mediated photobleaching that would report the extent of virus vector movement in the infected plants. All constructs had the inserted sequence placed into the multiple cloning site of the PVX vector pGR107 (Fig. 1A) and they were in Ti plasmid vectors so that *Agrobacterium* infiltration could be used for virus inoculation. Inoculation of Nb was a positive control that has been well characterized with the PVX vector system.

At 21 DAI with *PVX::PDS*, photobleaching was observed due to silencing of *PDS* in both species (Fig. 1B; *Nb* and *Sly* leaves, *PVX::PDS* panels) but not in the controls (Fig. 1B; *Nb* and *Sly* leaves, non-inoculated and *PVX::EV* panels). *PDS* transcript levels assayed by qRT–PCR were lower in the photobleached leaves of the *PVX::PDS*-inoculated plants than in the controls (Fig. 1C, D). Photobleaching of tomato fruits was also observed with *PVX:PDS* inoculation (Fig. 1B; *Sly* fruit, *PVX::PDS* panels), indicating that the PVX vector was persistent in the inoculated plants until fruit ripening. These results confirmed that the PVX vector is functional in tomato.

*PVX::FT* and *PVX::SFT* were inoculated onto tomato cotyledons at 14 DAG to investigate the potential of this system for VIF. The inoculated plants developed normally with mild *PVX*-induced systemic symptoms (Fig. 1E), and there was accumulation of *PVX* RNA (Fig. 1F). Furthermore, qRT–PCR analysis confirmed VOX because *FT* and *SFT* RNA were more abundant in plants inoculated with the respective PVX vectors (Fig. 1G, H). There was, however, a lower *FT* overexpression level with the *SFT* construct.

### PVX-induced *FT* and *SFT* expression accelerates flowering and fruit production in tomato

**Fig. 2.**
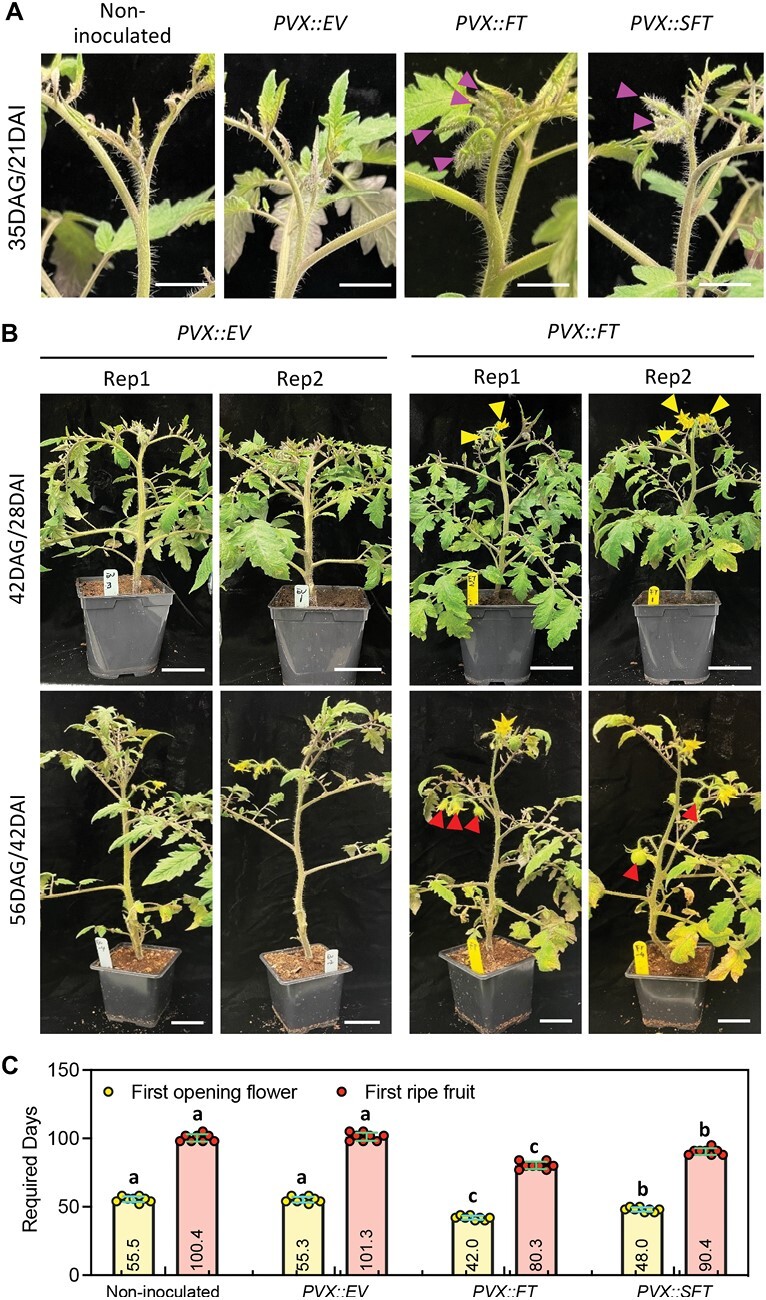
PVX-based VOX of FT-accelerated flowering and fruiting in tomato. (A) Apical shoots of non-inoculated and *PVX::EV-, PVX::FT-* and *PVX::SFT-*inoculated tomato plants at 21 DAI. Scale bars=1 cm. (B) *PVX::EV-* and *PVX::FT-*inoculated plants at 28 DAI and 42 DAI. Rep1 and Rep2 are two repetitions from two independent plants. Scale bars=2 cm. (C) Timing of first flower and first ripe fruit on non-infected and *PVX::EV-*infected controls or *PVX::FT- PVX::SFT-*infected plants. Values are means ±SD and the average days required are shown on the bottom of each bar (*P*<0.05, Student’s *t*-test). Eight tomato plants were used for each group and the experiment was repeated three times.

Both PVX and PVX-expressed *FT* should be transported towards the shoot apical meristem (Hein *et al.*, 2005; Jaeger and Wigge, 2007) and, consistent with effects on flowering, the inflorescences appeared earlier and the number of floral buds were greater on plants infected with *PVX::FT* and *PVX::SFT* than on the controls (Fig. 2A, pink arrowheads). The open flowers appeared consistently at 42–48 DAG rather than 55 DAG (Fig. 2B, yellow arrowheads) and the ripe fruits appeared at 80–90 DAG rather than 100 DAG or longer in the controls (Fig. 2C). Consistent with the VOX of *FT* (Fig. 1G, H), the acceleration was greater with *PVX::FT* rather than *PVX::SFT* (Fig. 2C).

**Fig. 3.**
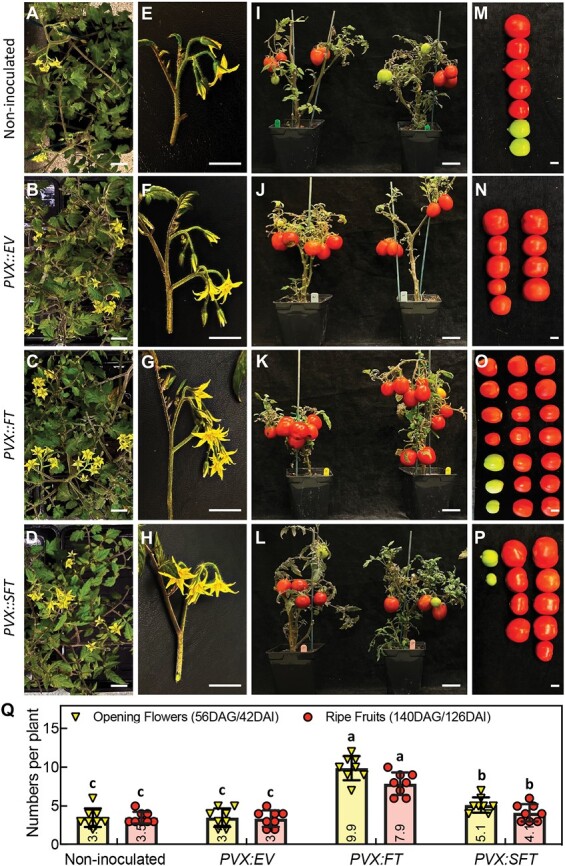
*PVX* induced *FT* expression increased flower numbers and fruit yield in tomato. (A-D) Top view of (A) non-inoculated; (B) *PVX:EV*; (C) *PVX:FT*; and (D) *PVX:SFT* plants at 42 DAI. Scale bars=2 cm. (E-H) A single inflorescence of (E) non-inoculated; (F) *PVX:EV*; (G) *PVX:FT*; and (H) *PVX:SFT* plants at 42 DAI. Scale bars=2 cm. (I-L) Plants that were (I) non-inoculated or inoculated with (J) *PVX:EV*; (K) *PVX:FT*; and (L) *PVX:SFT* at 126 DAI. Scale bars=5 cm. (M-P) Fruits from plants in (I)-(L); scale bars=2 cm. (Q) Number of open flowers and ripe fruits in non-inoculated, *PVX:EV*, *PVX:FT,* and *PVX:SFT* plants at 42 and 126 DAI stages, respectively. Values are means ±SD and the average numbers of flowers or fruits are shown on the bottom of each bar (*P*<0.05, Student’s *t*-test). Eight tomato plants were used for each group and the experiment was repeated three times.

The number of flowers and fruits also increased in the *PVX::FT*- and *PVX::SFT*-infected tomato plants. At 42 DAI, the plants infected with the *FT* constructs had 100% (*PVX::FT*) or 87.5% (*PVX::SFT)* more open flowers than the controls (Fig. 3A-H, Q). By 126 DAI the plants infected with the *FT* constructs had 100% (*PVX::FT*) or 62.5% (*PVX::SFT)* more fruits than the controls (Fig. 3I-P, Q). These findings validate the *PVX*-induced expression of *FT* with FT proving more effective than SFT in all of our assays. We cannot rule out a protein-mediated basis for this difference but given the difference in *FT* RNA levels (Fig. 1G, H) a more likely explanation invokes a VIGS effect in which an *SFT* but not *FT* viral insert silences the endogenous *SFT* RNA.

**Fig. 4.**
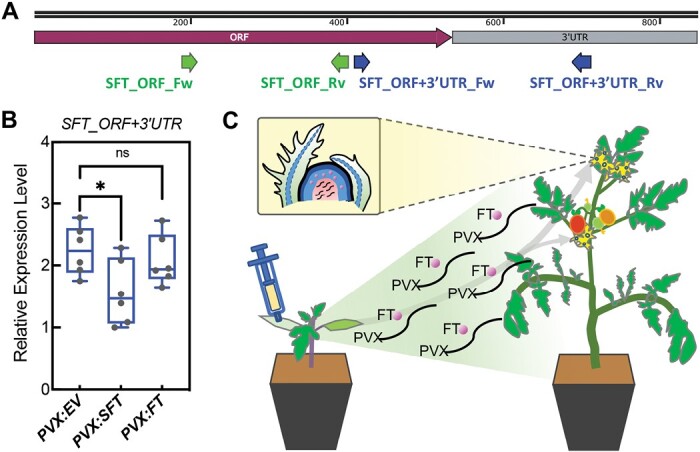
Endogenous *SFT* gene in the host tomato plant is partially silenced when expressing *PVX::SFT.* (A) Schematic diagram of *SFT* cDNA sequence and the primers used for testing expression level of overexpressing *SFT* from PVX vector (green) and endogenous *SFT* gene (blue) in the host tomato plant. (B) Relative expression level of *SlySFT_3’UTR* in *PVX::EV-*, *PVX::SFT-,* and *PVX::FT-*inoculated tomato leaves at 21 DAI determined by qRT–PCR (*P<0.05, Student’s *t*-test; *n*=6). The box plot shows minimum to maximum with all points. (C) Working model of PVX-induced *FT* expression. The PVX vector carrying FT was infiltrated in the tomato cotyledons and expressed in the systemic leaves along with the PVX virus. This allows the FT protein to move from leaves to the stem apex to facilitate specific cells to transform into flower primordium. The enlarged box indicates the shoot apical meristem (SAM) region in *PVX::FT* inoculated tomato. The virus usually cannot pass through into the SAM, however FT proteins encoded from the *PVX::FT* recombinant vector can.

To explore this possibility, we designed RT–PCR primers from the ORF and 3ʹ UTR that would amplify cDNA corresponding to the endogenous rather than viral *FT* RNA (Fig. 4A). The results showed that *SFT* endogenous transcript decreased in *PVX::SFT*-inoculated leaves more than with *PVX:EV* or *PVX:FT* (Fig. 4B). It is therefore likely that VIGS of the endogenous *FT* RNA in *PVX::SFT*-inoculated leaves resulted in lower levels of FT compared with those inoculated with *PVX:FT*.

**Fig. 5.**
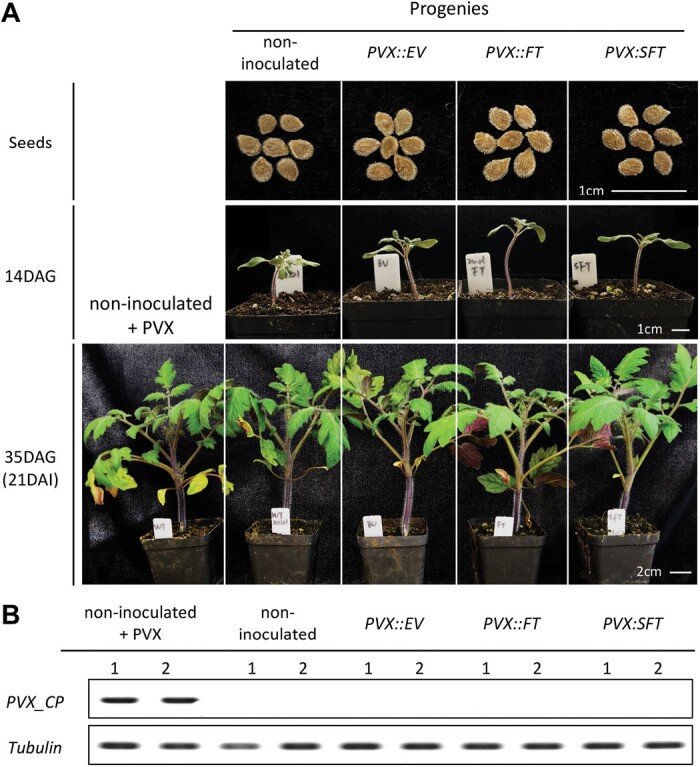
PVX virus was not detected in the progeny of PVX-infected tomato plants. (A) Seeds (top), 14 DAG seedlings (middle), and 35 DAG plants (bottom) of inoculated and non-inoculated tomato plants, with a group of non-inoculated progenies being inoculated with PVX for 21 d (bottom and left). Eight tomato plants were used for each group and the experiment was repeated three times. (B) Abundance of PVX coat protein (PVX_CP) of tomato plants in (A, bottom) determined by RT–PCR; each group included two biological replicates (1 and 2).

The VOX effect did not persist into the next generation. Seedling progeny of the infected plants (Fig. 5A) did not have viral symptoms or detectable viral RNA (Fig. 5B). We are thus confident that VIF-mediated speed breeding could be used in tomato without the possibility of seed transmission of the PVX vector.

Our findings advance previous technology in two ways. Firstly, they validate the use of PVX as an alternative to the TRV vector system for use in tomato (Brigneti *et al.*, 2004). PVX has a mono- rather than bi-partite viral RNA genome and may be simpler to use in many contexts. Secondly, the reduced endogenous RNA silencing resulting from the use of a heterologous gene for overexpression (Fig. 4) is an advantage over previous methods. This strategy to avoid RNA silencing of endogenous genes had a big effect on the overexpression of *FT* (Figs 2, 3) and may also be generally relevant when using virus vectors for gene overexpression.

## Supplementary data

The following supplementary data available at *JXB* online.

Table S1. Primers used in this study.

Fig. S1. Pairwise sequence alignment between Arabidopsis FT and tomato SFT.

erad369_suppl_Supplementary_Tables_S1_Figures_S1

## Data Availability

All data supporting the findings of this study are available within the paper and within its supplementary data published online.
