# Supplementary material for: Virus-induced overexpression of heterologous FLOWERING LOCUS T for efficient speed breeding in tomato
Source: J Exp Bot. 2023 Oct 3;75(1):36–44. doi: 10.1093/jxb/erad369 (PMC10735598; doi:10.1093/jxb/erad369)
Supplement: erad369_suppl_Supplementary_Tables_S1_Figures_S1 [file erad369_suppl_supplementary_tables_s1_figures_s1.pdf]

**Supplementary Fig. S1. Pairwise Sequence Alignment between Arabidopsis FT and tomato**

**SFT** CDS sequence:

```
AtFT_PEP           1 MSIN-IRDPLIVSRVVGDVLDPFNRSI-TLKVTYGQ-REVTNGLDLRPSQ     47
                     |. .  ||||:|.||||||||||.|:|  |:|.| : |||.||.:|||||
SlySFT_PEP         1 MP-RE-RDPLVVGRVVGDVLDPFTRTIG-LRVIY-RDREVNNGCELRPSQ     46

AtFT_PEP          48 V-QNKPRVEIGGEDLRNFYTLVMVDPDVPSPSNPHLREYLHWLVTDIPAT     96
                     |  |:||||:||:|||.|:||||||||.||||:|:|||||||||||||||
SlySFT_PEP        47 VI-NQPRVEVGGDDLRTFFTLVMVDPDAPSPSDPNLREYLHWLVTDIPAT     95

AtFT_PEP          97 TGTTFGNEIVCYENPSPTAGIHRVVFILFRQLGRQTVYAPGWRQNFNTRE    146
                     ||::||.|||.||:|.|:.||||.||:||||||||||||||||||||||:
SlySFT_PEP        96 TGSSFGQEIVSYESPRPSMGIHRFVFVLFRQLGRQTVYAPGWRQNFNTRD    145

AtFT_PEP         147 FAEIYNLGLPVAAVFYNCQRESGCGGRR--L-    175
                     |||:||||||||||::|||||||.||||  .
SlySFT_PEP       146 FAELYNLGLPVAAVYFNCQRESGSGGRRRSAD    177

# Program:      Pairwise Sequence Alignment (Needleman-Wunsch)
# Identity:     134/182 (73.6%)
# Similarity:   154/182 (84.6%)
```

Protein sequence:

```
AtFT_CDS           1 ATGTCTATAAATATAAGAGACCCTCTTATAGTAAGCAGAGTTGTTGGAGA     50
                     |||.|||.|.|   |.|.||.||||||.|.||..|..|.||.||.||.||
SlySFT_CDS         1 ATGCCTAGAGA---ACGTGATCCTCTTGTTGTTGGTCGTGTGGTAGGGGA     47

AtFT_CDS          51 CGTTCTTGATCCGTTTAATAGATCAATCACTCTAAAGGTTACTTATGGCC    100
                     .||..|.||.||.||.|..|||.|.||....||||..||||..|||.|..
SlySFT_CDS        48 TGTATTGGACCCTTTCACAAGAACTATTGGCCTAAGAGTTATATATAGAG     97

AtFT_CDS         101 AAAGAGAGGTGACTAATGGCTTGGATCTAAGGCCTTCTCAGGTTCAAAAC    150
                     |.|||||.||.|.||||||.|..||.||||||||.||.|||...|||
SlySFT_CDS        98 ATAGAGAAGTTAATAATGGATGCGAGCTTAGGCCTTCCCAAGTTATTAAC    147

AtFT_CDS         151 AAGCCAAGAGTTGAGATTGGTGGAGAAGACCTCAGGAACTTCTATACTTT    200
                     .|||||||.|||||..||||.|||||.|||||..|.|.|||.|..|||||
SlySFT_CDS       148 CAGCCAAGGGTTGAAGTTGGAGGAGATGACCTACGTACCTTTTTCACTTT    197

AtFT_CDS         201 GGTTATGGTGGATCCAGATGTTCCAAGTCCTAGCAACCCTCACCTCCGAG    250
                     ||||||||||||.||.||||.|||||||||.||..|.||..|.||..|||
SlySFT_CDS       198 GGTTATGGTGGACCCTGATGCTCCAAGTCCGAGTGATCCAAATCTGAGAG    247

AtFT_CDS         251 AATATCTCCATTGGTTGGTGACTGATATCCCTGCTACAACTGGAACAACC    300
                     ||||.||.||.||||||||.||.|||||.||.|||||.||.||..|||..
SlySFT_CDS       248 AATACCTTCACTGGTTGGTCACCGATATTCCAGCTACCACAGGTTCAAGT    297

AtFT_CDS         301 TTTGGCAATGAGATTGTGTGTTACGAAAATCCAAGTCCCACTGCAGGAAT    350
                     |||||..|.||.||.|||.|.||.||||.||||||.||..|....|||||
SlySFT_CDS       298 TTTGGGCAAGAAATAGTGAGCTATGAAAGTCCAAGACCATCAATGGGAAT    347

AtFT_CDS         351 TCATCGTGTCGTGTTTATATTGTTTCGACAGCTTGGCAGGCAAACAGTGT    400
                     .|||||..|.||.|||.||||.||..||||..|.||..||||||||||||
SlySFT_CDS       348 ACATCGATTTGTATTTGTATTATTCAGACAATTAGGTCGGCAAACAGTGT    397

AtFT_CDS         401 ATGCACCAGGGTGGCGCCAGAACTTCAACACTCGCGAGTTTGCTGAGATC    450
                     ||||.|||||.|||||.|||||.||||||||..|.||.|||||.||..|.
SlySFT_CDS       398 ATGCTCCAGGATGGCGTCAGAATTTCAACACAAGAGATTTTGCAGAACTT    447

AtFT_CDS         451 TACAATCTCGGCCTTCCCGTGGCCGCAGTTTTCTACAATTGTCAGAGGGA    500
                     ||.|||||.||..|.||.||.||.||.||.|..|..||||||||.||.||
SlySFT_CDS       448 TATAATCTTGGTTTACCTGTTGCTGCTGTCTATTTTAATTGTCAAAGAGA    497

AtFT_CDS         501 GAGTGGCTGCGGAGGA---AGAAGA-CT-----TTAG    528
                     |||||||.|.||.|||   |||||| ||     ||..
SlySFT_CDS       498 GAGTGGCAGTGGTGGACGTAGAAGATCTGCTGATTGA    534

# Program:      Pairwise Sequence Alignment (Needleman-Wunsch)
# Identity:     372/537 (69.3%)
# Similarity:   372/537 (69.3%)
```

**Supplementary Table S1. Primers used in this study**

| ID | Sequence | Usage |
|---|---|---|
| YD001 | AACCCGGGGTCATGCCTAGAGAACGTGAT | Vector construction: pGR107:SlySFT_FP |
| YD002 | TTCCCGGGTCAATCAGCAGATCTTCTACG | Vector construction: pGR107:SlySFT_RP |
| YD003 | AACCCGGGATGTCTATAAATATAAGAGACC | Vector construction: pGR107:AtFT_FP |
| YD004 | TTCCCGGGCTAAAGTCTTCTTCCTCCGCAG | Vector construction: pGR107:AtFT_RP |
| YD015 | CTAGCATCGATTCCCTGCCGGACAGGGTGACAGA | Vector construction: pGR107:PDS_FP |
| YD016 | ATCGGCGGTCGACCCGCGGCAAACACAAAAGCATCT | Vector construction: pGR107:PDS_RP |
| YD023 | CAGCACCAGCTAGCACAACA | PCR PVX_CP FP |
| YD024 | GACAGCATTGCTGGCTACTATG | PCR PVX_CP RP |
| YD025 | AGGTTATTCTAGGACTTCCAAATCT | PCR PVX_25k FP |
| YD026 | TGCCTCTAGTTCTGATACTCACCT | PCR PVX_25k RP |
| YD029 | AACCTCCATTCAGGAGATGTTT | qPCR ref_Tubulin_FP |
| YD030 | TCTGCTGTAGCATCCTGGTATT | qPCR ref_Tubulin RP |
| YD041 | CTATACTTTGGTTATGGTGGA | qPCR FT qRT-PCR FP |
| YD042 | GCATACACTGTTTGCCTGCCAA | qPCR FT qRT-PCR RP |
| YD043 | TTTCACTTTGGTTATGGTGGA | qPCR SFT qRT-PCR FP |
| YD044 | GCATACACTGTTTGCCGACCTA | qPCR SFT qRT-PCR RP |
| YD199 | GGCGTCAGAATTTCAACACA | qPCR SFT_cDNA_qPCR_FP |
| YD204 | AAGTCAGTCTTTGTTTCTTTATTGA | qPCR SFT_cDNA_qPCR_RP |
| YD207 | TCAGGGCACCCTCTTCGATCTGATCCT | qPCR ref_SKP1_FP |
| YD208 | TGTCTGCCACGGTCTGGCAAGTGA | qPCR ref_SKP1_RP |
